# Overexpression of PTPRZ1 Regulates p120/*β*-Catenin Phosphorylation to Promote Carcinogenesis of Oral Submucous Fibrosis

**DOI:** 10.1155/2022/2352360

**Published:** 2022-02-23

**Authors:** Liwei Ma, Ting Shen, Hui Peng, Jianjun Wu, Wenjin Wang, Xing Gao

**Affiliations:** ^1^Department of Oral Medicine, Center of Stomatology, Xiangya Hospital, Central South University, Changsha, Hunan Province 410008, China; ^2^Department of Oral and Maxillofacial Surgery, Center of Stomatology, Xiangya Hospital, Central South University, Changsha, Hunan Province 410008, China; ^3^Center of Oral and Maxillofacial Cancer (COMAC), Xiangya Hospital, Central South University, Changsha, Hunan Province 410008, China; ^4^Institute of Oral Cancer and Precancerous Lesions, Central South University, Changsha, Hunan Province 410008, China; ^5^Xiangya Stomatological Hospital, Xiangya School of Stomatology, Central South University, Changsha, Hunan Province 410008, China; ^6^Key Laboratory of Molecular Radiation Oncology Hunan Province, Changsha, Hunan Province 410008, China; ^7^National Clinical Research Center for Geriatric Disorders, Xiangya Hospital, Central South University, Changsha, Hunan 410008, China

## Abstract

**Background:**

Oral submucous fibrosis (OSF) is a potentially malignant disease of the oral cavity. New molecular predictors are needed to identify the high risk of malignant transformation in potentially malignant oral lesions. Our purpose is to explore PTPRZ1 and p120/*β*-catenin pathogenesis in the carcinogenesis of OSF to identify novel drug targets.

**Methods:**

The expression of PTPRZ1, p120, and *β*-catenin in clinical tissues was detected. Then, PTPRZ1, p120, *β*-catenin, RhoA, Rac1, CDC42, cyclin D1, and c-myc expressions were detected by qRT-PCR and western blot. CCK-8 was applied to measure hOMF cells viability. Wound healing and transwell assay were applied to measure cell migration and invasion. Western blot and IF detected the distribution of p-p120 and p-*β*-catenin. Tumor formation experiment explored PTPRZ1 effects on OSF.

**Results:**

PTPRZ1, p120, and *β*-catenin were abnormally expressed in cancer tissues. PTPRZ1 regulated the phosphorylation of p120/*β*-catenin. Western blot and IF showed that in the oe-NC group, p-p120 and p-*β*-catenin were expressed in the cell membrane. p-p120 and p-*β*-catenin were expressed in the cytoplasm and nucleus of the oe-PTPRZ1 group. *In vitro* experimental results revealed overexpression of PTPRZ1 and *β*-catenin, and silencing of p120 promoted cell proliferation, migration, and invasion. The tumor volume and weight in the sh-PTPRZ1 group were significantly reduced. IHC revealed the positive rate of PTPRZ1 was also low.

**Conclusions:**

Overexpression of PTPRZ1 regulated the phosphorylation of p120/*β*-catenin to promote OSF malignancy.

## 1. Introduction

Oral submucous fibrosis (OSF) is a chronic, insidious, and progressive oral mucosal disease [1, 2]. It is characterized by abnormal collagen deposition, which can produce scars and tissue fibrosis [3]. OSF is a common oral precancerous lesion in Asian countries, especially in areas where the culture of areca nut chewing is prevalent. The age of illness is between 20 and 40 years old [4]. The patient has submucosal fibrosis, ulcers, dry mouth, burning sensation, and restricted mouth opening, which greatly affects the quality of life of the patient [3]. Among them, about 1.5%–15% of all cases are transformed into malignant tumors [5]. It was reported that the incidence of oral squamous cell carcinoma (OSCC) caused by chewing areca is high in Asia-Pacific countries [6]. In order to reduce OSF occurrence, early detection of premalignant diseases, understanding the mechanism of OSF induction and transformation, and taking active therapeutic interventions are very important. Although many OSF lesions biomarkers have been identified and different drug combinations trials have been carried out, clinicians still adopt conservative treatments, mainly focusing on alleviating OSF symptoms. Therefore, new molecular predictors are needed to identify the high risk of malignant transformation in potentially malignant oral lesions.

Receptor-type tyrosine-protein phosphatase zeta (PTPRZ1) is a member of the RPTP family [7]. PTPRZ1 has different functions in the occurrence, development, and metastasis of cancer, which further affects cancer treatment and prognosis. Studies have found that PTPRZ1 is abnormally expressed in various types of tumors. PTPRZ1 is upregulated in human astrocytomas, cervical carcinoma, and small-cell lung carcinoma [8–10]. It is downregulated in breast invasive carcinoma and stomach adenocarcinoma [11]. Some studies also have shown that PTPRZ1 plays a vital role in the occurrence, development, invasion, and metastasis of OSF carcinogenesis and can be used as a molecular marker for early diagnosis and a target gene for treatment [12]. It has been confirmed that PTPRZ1/*β*-catenin is an important pathway that regulates tumorigenesis. PTPRZ1 can enhance *β*-catenin protein expression in the nucleus of human renal cell carcinoma and participates in regulating proliferation by activating *β*-catenin and its downstream genes [13].

Catenin cell-cell adhesion complex plays a vital role in the development and progression of cancer [14]. p120-catenin (p120) is a cytoplasmic molecule closely related to E-cadherin. P120 coexists with *β*-catenin or *γ*-catenin in the E-cadherin complex and plays a vital role in stabilizing the E-cadherin-catenin complex [15]. The expression of p120 has been extensively studied in many human cancers. Studies have shown that the expression of phosphorylated p120-catenin has predictive value for the progression of oral cancer [16]. In addition, phosphorylation of p120 is an effective substrate of the oncogenic Src family, and its phosphorylation is related to cell transformation [17]. *β*-Catenin is a protein containing a central armadillo (ARM) domain (residues 140–664), amino-terminus domain (NTD), and carboxyl terminus domain (CTD) [18]. It is encoded by the CTNNB1 gene and is a dual-function protein that regulates the coordination of cell-cell adhesion and gene transcription [19, 20]. Abnormal expression of *β*-catenin is related to metastatic oral squamous cell carcinoma (OSCC) with positive cervical lymph nodes [21]. However, little is known about the relationship between OSF and p120/*β*-catenin.

Therefore, we hypothesized that the PTPRZ1/*p*120/*β*-catenin axis might play a role in the malignant transformation of OSF. We verified that OSF malignant transformation is related to the PTPRZ1/*p*120/*β*-catenin axis in human tissues. In addition, we used arecoline-induced cell models to elucidate the mechanism of PTPRZ1 regulated the phosphorylation of p120/*β*-catenin to promote OSF malignant transformation and to clarify its value as a potential therapeutic target, so as to provide novel ideas and theoretical evidence for the prevention and treatment of OSF malignant transformation.

## 2. Materials and Methods

### 2.1. Clinical Tissue Samples

Forty participants were recruited from Xiangya Hospital of Central South University. Human normal oral mucosal tissue, early OSF tissue, middle OSF tissue, and OSCC tissue were collected. The groups were normal group, early-OSF group, medium-OSF group, and the OSCC group. We have obtained the written informed consent of the subjects before the start of the study and obtained the approval of the Medical Ethics Committee of Xiangya Hospital (No. 201703635).

### 2.2. Cell Culture and Treatment

Normal oral epithelial cells (HNOEC cells), OSCC cells (CAL27 cells), and human oral mucosa fibroblasts (hOMF cells) were purchased from the Cell Bank of the Chinese Academy of Sciences. HNOEC cells were cultured in a Minimum Essential Medium containing 10% FBS (Thermo Fisher Scientific). CAL27 cells were cultured in DMEM containing 10% FBS and 1% penicillin/streptomycin (P/S, C0222, Beyotime Biotechnology). hOMF cells were cultured in 90% H-DMEM and 10% FBS containing 100 U/mL P/S medium. They were placed at 37°C, cultivated in a humidity chamber with 5% CO_2_. They were divided into the control (HNOEC cells), OSCC (CAL27 cells), OSF (hOMF cells), OSF + arecoline (hOMF cells), oe-NC (hOMF cells), and oe-PTPRZ1 (hOMF cells) groups. The OSF + arecoline group was treated with 0.2 *μ*g/mL arecoline for 24 h in hOMF cells [22]. In order to overexpress PTPRZ1, the PTPRZ1 sequence was linked to the LV003 vector. According to the instructions, the PTPRZ1-vector plasmid was transfected into hOMF cells using lipofectamine 3000 reagent. The oe-NC group was transfected with oe-NC plasmid in hOMF cells, and the oe-PTPRZ1 group was transfected with PTPRZ1-vector plasmid in hOMF cells. The shRNA-1 targeting human p120 (5′-GATAGAAGCTGAGGCTGAAGTTGAA-3'), shRNA-2 targeting human p120 (5′-GGATGCTGTTAGACCTTCTACTTGA-3′), and shRNA-3 targeting human p120 (5′-CGATCCTTAACATTGAGGCTGATAA-3') synthesized by Sangon Biotech (Shanghai, China) and the corresponding negative control sh-NC (5′-UUCUCCGAACGUGUCACGUTT-3′) were used to knock down the expression of p120. They were divided into the sh-NC, sh-p120-1, sh-p120-2, and sh-p120-3 groups. In order to overexpress *β*-catenin, the *β*-catenin sequence was linked to the vector LV003. According to the instructions, *β*-catenin-vector plasmid was transfected into hOMF cells using lipofectamine 3000 reagent. They were divided into the oe-NC group and the oe-*β*-catenin group.

### 2.3. Quantitative Real-Time PCR (qRT-PCR)

QRT-PCR was performed to detect PTPRZ1, p120, *β*-catenin, RhoA, Rac1, CDC42, cyclin D1, and c-myc expression levels. To put it simply, total RNA was extracted by the Trizol method, RNA was reversely transcribed into cDNAs in accordance with the instruction of a reverse transcription kit (CW2569, CWBIO, China). SYBR Green qPCR mix (Invitrogen) was performed to test genes relative expression in ABI 7900 system. The relative level of the gene was calculated by the 2^–ΔΔCt^ method with GAPDH as the internal gene. The primer sequence used in this study is shown in Table 1. Each group was tested three times.

### 2.4. Western Blot (WB)

RIPA lysis buffer (#P0013 B, Beyotime Biotechnology) was applied to extract the total protein from cells and tissues according to the instructions. The protein of each group was quantified according to the BCA protein determination kit, and the SDS-PAGE loading buffer (#MB2479, Meilunbio) was mixed; the mixture was heated for 5 minutes in boiling water at 100°C. The protein was adsorbed on the PVDF membrane by gel electrophoresis, and then it was sealed at 2 hours with a 5% skim milk solution at room temperature. PTPRZ1 (55125-1-AP, 1:1,000, Proteintech), p120 (12180-1- AP, 1:1,000, Proteintech), p-p120 (ab173578, 1:20,000, Abcam), *β*-catenin (51067-2-AP, 1:10,000, Proteintech), p-*β*-catenin (ab27798, 1:500, Abcam), RhoA (10749-1-AP, 1:1,000, Proteintech), Racl (24072-1-AP, 1:1,000, Proteintech), CDC42 (10155-1-AP, 1:1,000, Proteintech), cyclin D1 (26939-1-AP, 1:1,000, Proteintech), C-myc (10828-1-AP, 1:2,000, Proteintech) primary antibody, and *β*-actin (66009-1-Ig, 1:1,000, Proteintech) were incubated for 90 min at room temperature. At room temperature, TBST was washed three times, and the second antibody HRP goat anti-mouse IgG (SA00001-1, 1:5,000, Proteintech) and HRP goat anti-rabbit IgG (SA00001-2, 1:5,000, Proteintech) were incubated. After ECL exposure, Odyssey Infrared Imaging System (LI-COR Biosciences, Lincoln, NE, USA) was applied to detect the protein band, and the internal reference was *β*-actin. Each group was tested three times.

### 2.5. Immunohistochemistry (IHC)

The expression of PTPRZ1, p-p120, and *β*-catenin was detected by IHC in human and mouse tumor tissues. The slices were roasted at 60°C for 12 h; then the slices were dewaxed to water and heated to repair the antigen. A total of 1% periodic acid was added, and the endogenous enzyme was inactivated for 10 minutes at room temperature. PBS was washed 3 times for 3 minutes. PTPRZ1(55125-1-AP, 1:200, Proteintech), p-p120 (ab173578, 1:200, Abcam), and p-*β*-catenin (ab27798, 1:200, Abcam) primary antibodies were incubated overnight at 4°C, and PBS was washed 3 times for 5 minutes. The secondary antibody was incubated at 37°C for 30 min. PBS was washed 3 times for 5 minutes. We dropped the prepared color developing agent DAB working solution of 50–100 *μ*L, incubated for 5–10 min at room temperature, washed with distilled water, and blued them with PBS. All levels of alcohol (60–100%) were dehydrated for 5 min. After removal, it was placed in xylene for 10 min and then sealed with neutral gum and observed under a microscope. Each group was tested three times.

### 2.6. Immunofluorescence (IF)

IF detected the distribution of p-p120 and p-*β*-catenin. The slides were removed and washed with PBS 2–3 times. Then slides were fixed with 4% paraformaldehyde for 30 min and PBS was washed 3 times for 5 minutes. Then, it was permeabilized with 0.5% TritonX-100 at 37°C for 30 min. After washing with PBS, 5% BSA was sealed at 37°C for 1 h, p-p120 (ab173578, 1:50, Abcam) and p-*β*-catenin (ab27798, 1:50, Abcam) were incubated overnight at 4°C. PBS was washed 3 times for 5 minutes. Diluted CoraLite594-conjugated Goat Anti-Rabbit IgG (SA00013-4, 1:200, Proteintech) was added. Then they were incubated at 37°C for 90 min, and PBS was washed 3 times for 5 minutes. Then they were dyed with DAPI (Wellbio) at 37°C for 10 min. The plates were sealed and observed under a fluorescence microscope. Each group was tested three times.

### 2.7. Cell Counting Kit 8 (CCK-8) Assay

The cells in the logarithmic growth phase were seeded in 96-well plates, and each well was seeded with 1 × 10^4^ cells. Then, the cells were cultured in a 37°C, 5% CO_2_ incubator for 12 h, 24 h, 48 h, 72 h, 96 h, and 120 h, respectively. After experimental treatment, 10 *μ*L CCK-8 reagent was added in an incubator for 2 h. The microplate reader (Infinite M200, Tecan, Austria) was performed to measure absorbance at 450 nm to assess cell viability in each group. Each group was tested three times.

### 2.8. Wound Healing

When the cells grew at a fusion rate of 90%, the cells were washed once with sterile PBS, scratched with a pipette tip, and washed once with PBS. Then we removed the scratched cells and added serum-free DMEM high-sugar medium. Photographs were taken at 0 h and 24 h to detect cell migration. Each group was tested three times.

### 2.9. Transwell Assay

The cells were collected using the transwell system, resuspended with serum-free DMEM, and inoculated into transwell plate lumen. The inner side of the chamber on the transwell plate was precoated with Matrigel basement membrane matrix (BD Biocoat). The Matrigel glue was dissolved overnight at 4°C and then diluted with precooled basal medium (Matrigel: medium) at the ratio of 1:3. 40 µL of Matrigel glue was added to precooled transwell chamber and incubated at 37°C for 2 h to make Matrigel glue solidify. Cells were counted with basal medium and adjusted to 1 × 10^6^/mL, added 100 *μ*L to the upper chamber of the transwell chamber and 600 *μ*L of complete medium to the lower chamber. After incubating at 37°C for 24 h, the cells on the surface of the upper chamber were wiped with wet cotton swabs, fixed with 4% paraformaldehyde for 20 min, stained with 0.5% crystal violet for 5–10 min, and observed and photographed under a microscope. Each group was tested three times.

### 2.10. Cell Cycle Assay

The cell suspension was collected and centrifuged to obtain cell precipitate. PBS was washed 2–3 times to prepare single-cell suspension, and cells number was adjusted to 1 × 10^6^ cells/mL. A total of 150 *μ*L propidium iodide (PI) was added and stained at 4°C for 30 min in dark. PI was excited by a 488 nm argon-ion laser and received by a 630 nm pass filter. Ten thousand cells were collected through FSC/SSC scatter plot. The gating technique was used to eliminate adhesion cells and fragments, and the percentage of each cell cycle on the fluorescence histogram of PI was analyzed. Each group was tested three times.

### 2.11. Apoptosis Assay

The treated cells were digested and collected with trypsin without EDTA. Cells were washed with PBS twice and centrifuged at 2,000 rpm for 5 min each time; about 5 × 10^5^ cells were collected. We added 500 *μ*L of binding buffer to suspend cells. After adding 5 *μ*L Annexin V-FITC (KGA108, KeyGen) and mixing well, 5 *μ*L propidium iodide (PI) was added. The reaction time was 15 min at room temperature and kept away from light. Flow cytometry (A00-1-1102, Beckman) was performed within 1 h. Each group was tested three times.

### 2.12. EDU

According to the EDU detection kit (RiboBio), the proliferation rate of cells was detected. First, the EDU was marked. The EDU solution was diluted with cell medium at the ratio of 1,000:1 to prepare an appropriate amount of 50 *μ*m EDU culture medium. The cells were incubated overnight in 100 *μ*L 50 *μ*M EDU medium, and PBS was washed 2 times for 5 minutes. Then 50 *μ*L cell fixation solution (4% paraformaldehyde) was added, and it was incubated for 30 min at room temperature. After adding 50 *μ*L 2 mg/mL glycine and incubating in a decolorizing shaker for 5 min, PBS was washed for 5 minutes. Next, 100 *μ*L penetrant was added to decolorize, and it was incubated in a decolorizing shaker for 10 minutes. PBS was washed once for 5 minutes. We added 100 *μ*L of 1× Apollo® staining reaction solution, and it was incubated for 30 minutes in a decolorizing shaker at room temperature and kept away from light. Then we added 100 *μ*L of penetrant to decolorize and washed 2–3 times on decolorizing shaker for 10 minutes each time. After adding 100 *μ*L methanol to wash 1–2 times for 5 minutes each time and washing once with PBS for 5 minutes, DNA was stained and observed immediately after staining. Each group was tested three times.

### 2.13. *In Vivo* Tumorigenesis

Twelve SPF-grade, 4-week-old mice were randomly divided into OSCC and sh-PTPRZ1 group with 6 mice in each group. Animal studies were approved by the Medical Ethics Committee of Xiangya Hospital (No. 20173637). The short hairpin targeting PTPRZ1 (sh-PTPRZ1) synthesized by Sangon Biotech and corresponding negative control sh-NC were used to knock down PTPRZ1 expression. The OSCC group was injected with sh-NC treated OSCC cells, and the sh-PTPRZ1 group was injected with sh-PTPRZ1 treated OSCC cells, and they were injected into nude mice axilla [23]. The tumor volume of each group was measured at 4, 7, 11, 14, 17, 21, and 24 d. The nude mice were sacrificed at 24 days, and the tumor mass of each group was detected. The positive rate of PTPRZ1 in tumor tissues was detected by IHC.

### 2.14. Statistical Analysis

Graphpad 8.0 was applied for statistical analysis, and experimental data were expressed as mean ± SD, which was repeated at least 3 times. Pearson correlation coefficient analyzed the correlation between PTPRZ1 and p120, PTPRZ1 and *β*-catenin. One-way ANOVA was used for comparison between the two groups. *p* < 0.05 was considered statistically significant.

## 3. Results

### 3.1. Expression of PTPRZ1, p120, and *β*-Catenin in Tissues

**Figure 1:**
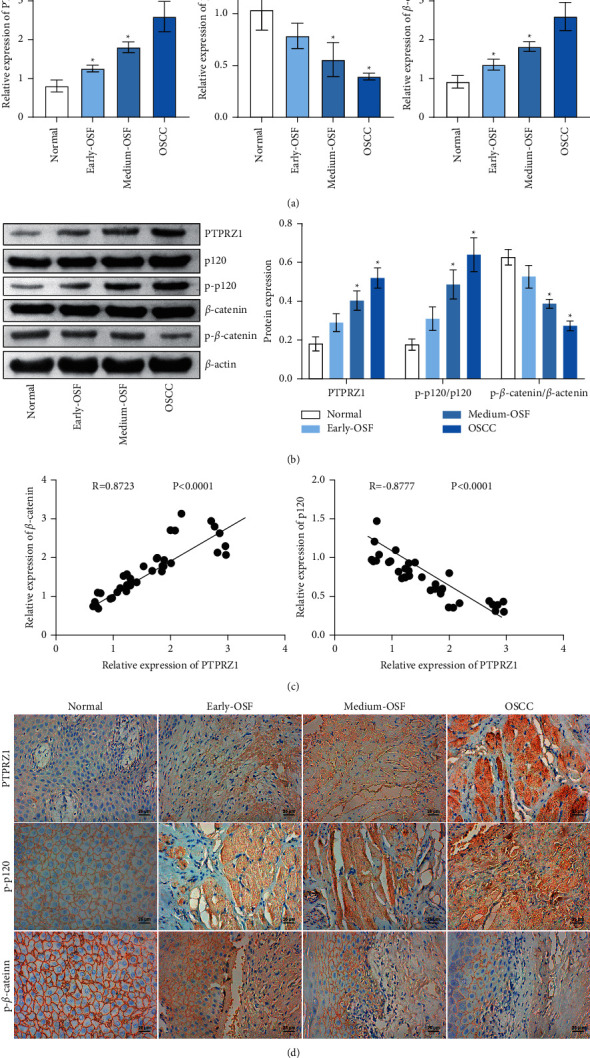
The expression of PTPRZ1, p120, and *β*-catenin in tissues. (a) qRT-PCR detected PTPRZ1, p120, and *β*-catenin levels in tissues (*n* = 3). (b) Western blot was performed to detect PTPRZ1, p-p120/*p*120, and p-*β*-catenin/*β*-catenin protein expression in tissues (*n* = 3). (c) Pearson correlation coefficient analyzed the correlation between PTPRZ1 and p120 and between PTPRZ1 and *β*-catenin. (d) IHC was performed to detect the positive rate of PTPRZ1, p-p120, and p-*β*-catenin in cancer tissues (*n* = 3).  ^*∗*^*p* < 0.05; scale bar = 25 µm; and the magnification is 400 times.

To investigate the expression of PTPRZ1, p120, and *β*-catenin in OSF tissues and OSCC tissues, qRT-PCR was first performed. The results revealed the expression of PTPRZ1 and *β*-catenin in early-OSF, middle-OSF, and OSCC groups increased compared with the normal group, while the expression of p120 decreased (Figure 1(a)). Western blot results revealed PTPRZ1, and p-p120/*p*120 expressions increased in the early-OSF, middle-OSF, and OSCC groups, while p-*β*-catenin/*β*-catenin decreased (Figure 1(b)). Pearson correlation coefficient analysis showed a positive correlation between PTPRZ1 and *β*-catenin and a negative correlation between PTPRZ1 and p120 (Figure 1(c)). The results of IHC revealed the positive rate of PTPRZ1, p-p120, and p-*β*-catenin in early-OSF, middle-OSF, and OSCC groups increased compared with the normal group, and the positive rate of p-*β*-catenin decreased (Figure 1(d)). Besides, p-*β*-catenin was mainly expressed in the cell membrane, but hardly in the cytoplasm and nucleus. PTPRZ1 and p-P120 were detected in all parts of the cells. The above results indicate that PTPRZ1, p120, and *β*-catenin are abnormally expressed in cancer tissues.

### 3.2. Overexpression of PTPRZ1 Regulated the Phosphorylation of p120/*β*-Catenin

**Figure 2:**
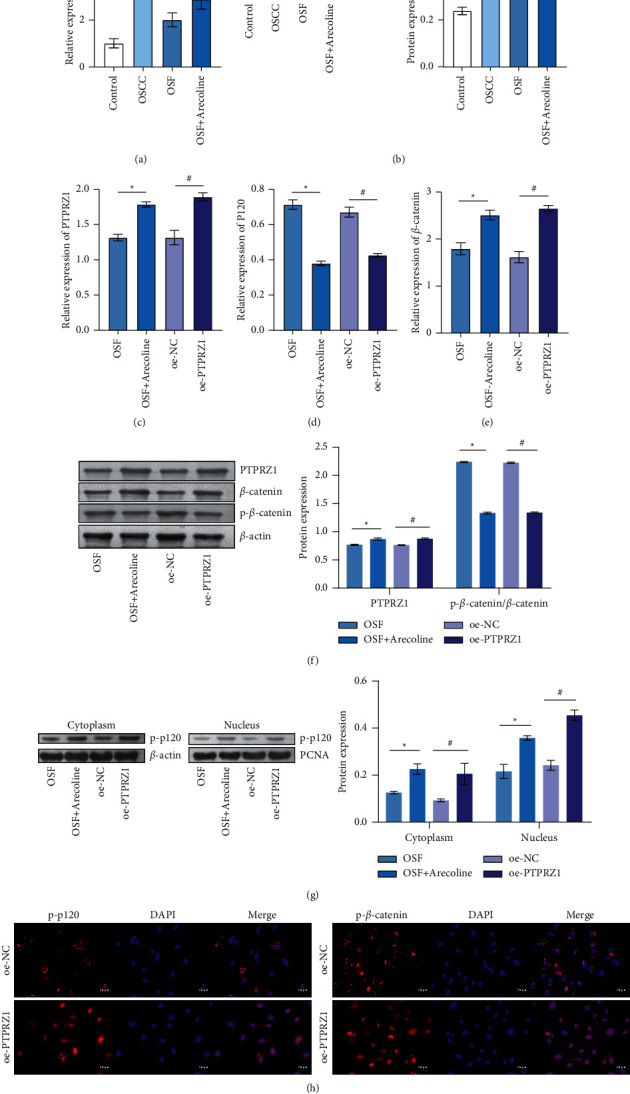
Overexpression of PTPRZ1 regulated the phosphorylation of p120/*β*-catenin. (a, b) qRT-PCR and western blot were used to detect PTPRZ1 expression in each group of cells (*n* = 3). *∗p* < 0.05 vs. control group (HNOEC cells). (c–e) qRT-PCR was performed to detect the expression of PTPRZ1, p120, and *β*-catenin in each group of cells (*n* = 3). (f) Western blot detected PTPRZ1, p120, and p-*β*-catenin/*β*-catenin protein expression in each group of cells (*n* = 3). (g) Western blot detected p-120 protein expression of cytoplasm or nucleus in each group of cells (*n* = 3). (h) IF detected the distribution of p-p120 and p-*β*-catenin in the oe-NC and oe-PTPRZ1 groups (hOMF cells, *n* = 3).  ^*∗*^*p* < 0.05 vs. the OSF group (hOMF cells); ^#^*P* < 0.05 vs. the oe-NC group (hOMF cells); scale bar = 25 µm; and the magnification is 400 times.

P120/*β*-catenin has been shown to affect the development of OSCC [14]. Next, we wanted to investigate whether PTPRZ1 affects OSF carcinogenesis by p120/*β*-catenin. PTPRZ1 mRNA and protein expressions in the OSCC group (CAL27 cells) were increased compared with the control group (HNOEC cells). Compared with the OSF group (hOMF cells), PTPRZ1 mRNA and protein expressions were increased in the OSF + arecoline group (Figures 2(a) and 2(b)). As shown in Figures 2(c)–2(g), PTPRZ1, p-p120, and *β*-catenin mRNA and protein expressions in the OSF + arecoline group were increased compared with the OSF group (hOMF cells), while the expressions of p-*β*-catenin and p120 were decreased. Compared with the oe-NC group (hOMF cells), PTPRZ1, p-p120, and *β*-catenin mRNA and protein levels were increased, and p-*β*-catenin and p120 expressions were decreased in the oe-PTPRZ1 (hOMF cells). At the same time, the content of p-P120 in the cytoplasm was higher than that in the nucleus, but the change trend in both was consistent. IF showed that p-p120 and p-*β*-catenin were expressed in the cell membrane in the oe-NC group (hOMF cells). In the oe-PTPRZ1 (hOMF cells), p-p120 and p-*β*-catenin were expressed in cytoplasm and nucleus (Figure 2(h)), These results revealed overexpression of PTPRZ1 regulated p120/*β*-catenin phosphorylation.

### 3.3. Overexpression of PTPRZ1 Promoted Oral Cell Carcinogenesis

**Figure 3:**
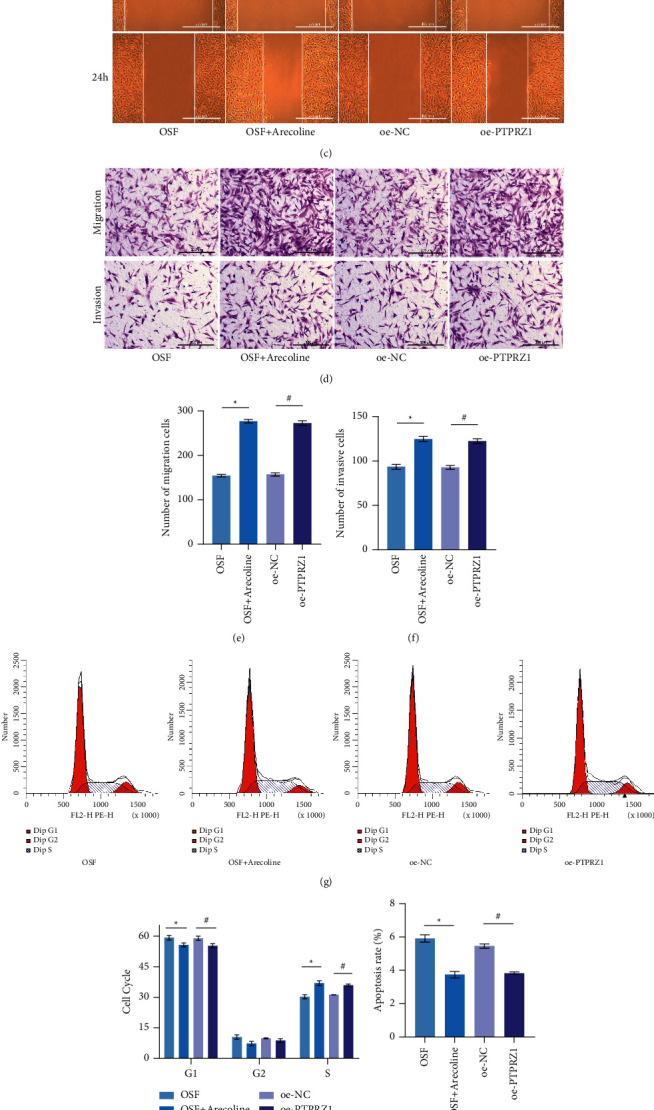
Overexpression of PTPRZ1 promoted cell proliferation, migration, and invasion. (a) CCK-8 detected the proliferation rate of cells in each group at 12 h, 24 h, 48 h, 72 h, 96 h h, and 120 h (*n* = 3). (b, c) Wound healing detected cell migration ability (*n* = 3). (d–f) Transwell assay detected the migration and invasion ability of cells in each group (*n* = 3). (g, h) Flow cytometry was applied to detect cell cycle in each group (*n* = 3). (i, j) Annexin V-FITC/PI flow cytometry detected the apoptosis rate of cells in each group (*n* = 3).  ^*∗*^*p* < 0.05 vs. the OSF group (hOMF cells);  ^#^*p* < 0.05 vs. the oe-NC group (hOMF cells); scale bar = 100 µm; and the magnification is 100 times.

From the above experiments, it was concluded that PTPRZ1 could regulate the expression of p120 and *β*-catenin. Then we want to further explore whether PTPRZ1 can promote OSF malignancy. As shown in Figure 3(a), compared with the OSF group (hOMF cells), the viability of the cells of the OSF + arecoline group (hOMF cells) after 12 h, 24 h, 48 h, 72 h, 96 h, and 120 h also increased. At the same time, the activity of the cells after 12 h, 24 h, 48 h, 72 h, 96 h, and 120 h of the oe-PTPRZ1 group (hOMF cells) were also increased compared with the oe-NC group (hOMF cells). Besides, compared with the OSF group (hOMF cells), the cells migration and invasion ability of the OSF + arecoline group (hOMF cells) also increased. The cells migration and invasion ability of the oe-PTPRZ1 group (hOMF cells) were also increased compared with the oe-NC group (hOMF cells; Figures 3(b)–3(f)). Compared with the OSF group (hOMF cells), the G0/*G*1 phase also decreased; the S phase increased; and apoptosis decreased in OSF + arecoline group. What is more, the G0/*G*1 phase decreased; the S phase increased; and apoptosis decreased in oe-PTPRZ1 (hOMF cells) compared with the oe-NC group (hOMF cells; Figures 3(g)–3(i)). These confirmed our conjecture that overexpression of PTPRZ1 promoted the carcinogenesis of OSF.

### 3.4. Knockdown of p120 Pathway Promoted Oral Cell Carcinogenesis

**Figure 4:**
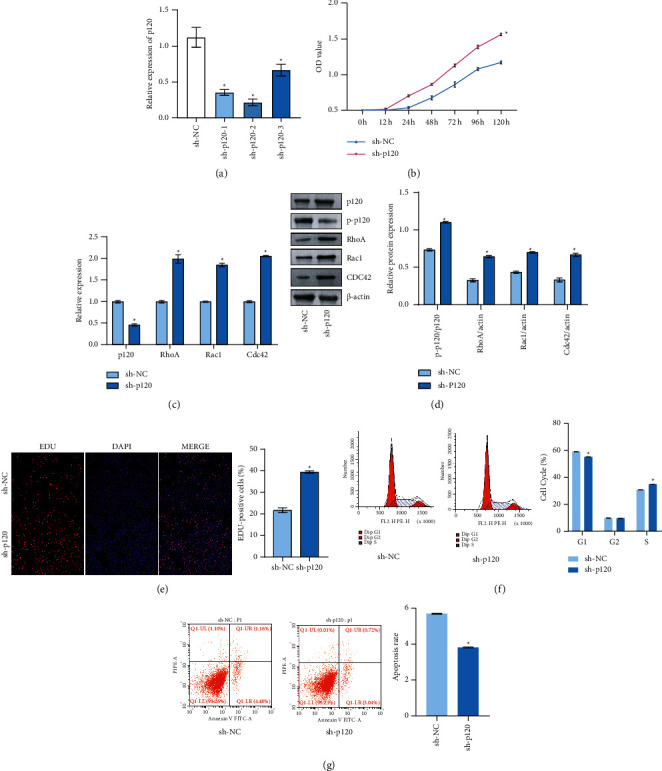
Knockdown of p120 pathway promoted oral cell carcinogenesis. (a) qRT-PCR was used to detect p120 expression in each group of cells.  ^*∗*^*p* < 0.05 vs. the sh-NC group (hOMF cells, *n* = 3). (b) CCK-8 detected the proliferation rate of cells in each group at 12 h, 24 h, 48 h, 72 h, 96 h, and 120 h (*n* = 3). (c) qRT-PCR detected p120, RhoA, Rac1, and CDC42 expressions (*n* = 3). (d) Western blot detected p-p120, p120, RhoA, Rac1, and CDC42 protein expression levels (*n* = 3). (e) EDU was performed to detect the proliferation rate of cells (*n* = 3). (f) Flow cytometry detected cell cycle in each group (*n* = 3). (g) Annexin V-FITC/PI flow cytometry was applied to detect the apoptosis rate of cells in each group (*n* = 3).  ^#^*p* < 0.05 vs. sh-NC group; scale bar = 100 µm; and the magnification is 100 times.

Next, we explored the effect of overexpression of p120 on OSF. Studies have shown that p120 aggregated to affect RhoA, Rac1, and CDC42 [24]. After we tried to treat hOMF cells with three kinds of sh-p120, the sh-p120-2 group with the most significant knockout effect was selected for follow-up study (Figure 4(a)), which was called the “sh-p120” group. As shown in Figure 4(b), the cell viability of the sh-p120 group (hOMF cells) increased compared with the sh-NC group (hOMF cells) after 12 h, 24 h, 48 h, 72 h, 96 h, and 120 h. As shown in Figures 4(c) and 4(d), p-p120, RhoA, Rac1, and CDC42 expressions in the sh-p120 group (hOMF cells) increased compared with the sh-NC group (hOMF cells), while p120 decreased. Compared with the sh-NC group (hOMF cells), the cell proliferation in the sh-p120 group (hOMF cells) increased; the S phase increased; the G0/*G*1 phase decreased; and the apoptosis decreased (Figures 4(e)–4(g)), indicating that the knockdown p120 promoted OSF cell carcinogenesis.

### 3.5. Activated *β*-Catenin Pathway Promoted Oral Cell Carcinogenesis

**Figure 5:**
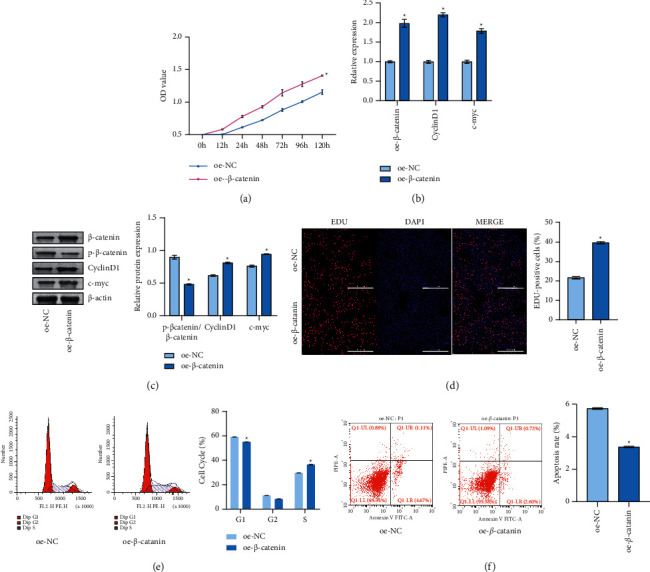
Activated *β*-catenin pathway promoted oral cell carcinogenesis. (a) CCK-8 detected the proliferation rate of cells in each group at 12 h, 24 h, 48 h, 72 h, 96 h, and 120 h (*n* = 3). (b) qRT-PCR was used to detect *β*-catenin, cyclin D1, and c-myc expressions (*n* = 3). (c) Western blot was used to detect *β*-catenin, p-*β*-catenin, cyclin D1, and c-myc protein expression levels (*n* = 3). (d) EDU detected the proliferation rate of cells in each group (*n* = 3). (e) Flow cytometry was performed to detect cell cycle (*n* = 3). (f) Annexin V-FITC/PI flow cytometry was used to detect the apoptosis rate of cells (*n* = 3).  ^*∗*^*p* < 0.05 vs. the oe-NC group (hOMF cells); scale bar = 100 µm; and the magnification is 100 times.

Then, we want to explore the effect of the expression of *β*-catenin pathway on OSF. Studies have shown that cyclin D1 and c-myc are involved in the occurrence, development, and promotion of cancer [25]. As shown in Figure 5(a), the cell viability of the oe-*β*-catenin group (hOMF cells) increased compared with the oe-NC group (hOMF cells) after 12 h, 24 h, 48 h, 72 h, 96 h, and 120 h. As shown in Figures 5(b) and 5(c), qRT-PCR and western blot detection results revealed compared with the oe-NC group (hOMF cells), cyclin D1, c-myc, and *β*-catenin increased in the oe-*β*-catenin group, while p-*β*-catenin expression decreased. Compared with the oe-NC group (hOMF cells), the cell proliferation in oe-*β*-catenin group increased; the S phase increased; the G0/*G*1 phase decreased; and the apoptosis decreased (Figure 5(d)–5(f)). It showed that activated *β*-catenin promoted OSF cell carcinogenesis.

### 3.6. Silencing PTPRZ1 Inhibited the Development of OSCC

**Figure 6:**
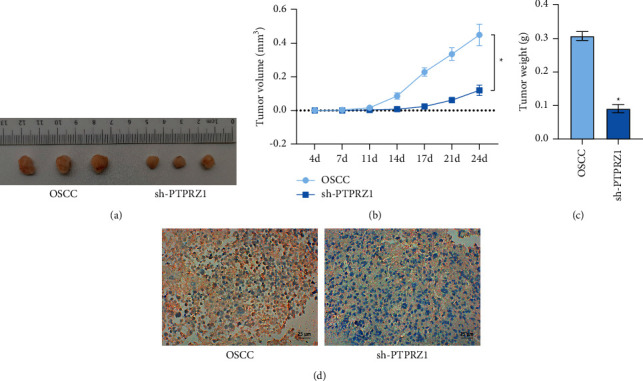
Silencing PTPRZ1 inhibited the development of OSCC. (a) Tumor formation in nude mice was observed by photographing (*n* = 3). (b) The tumor volume of nude mice in each group was measured at each time point of *t* 4 d, 7 d, 11 d, 14 d, 17 d, 21 d, and 24 d (*n* = 3). (c) The nude mice were sacrificed on 24 days, and the tumor weight of each group of nude mice was measured (*n* = 3). (d) PTPRZ1 positive rate in tumor tissue was detected by IHC (*n* = 3).  ^*∗*^*p* < 0.05 vs. the OSCC group (CAL27 cells); scale bar = 25 µm; and the magnification is 400 times.

In order to further investigate PTPRZ1 effects on OSCC, we performed nude mice tumorigenesis experiment and injected the OSCC cells treated with sh-PTPRZ1. As shown in Figures 6(c) and 6(d), the tumor volume in the sh-PTPRZ1 group was significantly reduced compared with the OSCC group. As shown in Figures 6(c) and 6(d), tumor weight was significantly reduced, and the positive rate of PTPRZ1 was lower. These results suggested that silencing PTPRZ1 inhibited the development of OSCC.

## 4. Discussion

OSF is a potentially malignant disease of the oral cavity. At present, conservative treatment to alleviate OSF is the main choice of clinical treatment, and there is a lack of good treatment methods for OSF. In this paper, we studied the possible role of the PTPRZ1/*p*120/*β*-catenin axis in the malignant transformation of OSF through clinical samples, arecoline-treated cell models, and nude mice tumorigenesis experiment. We found that the OSF malignant transformation in human tissues is related to the PTPRZ1/*p*120/*β*-catenin axis. In addition, PTPRZ1 could regulate the phosphorylation of the p120/*β*-catenin pathway to promote OSF malignancy.

PTPRZ1 is a transmembrane protein tyrosine phosphatase. Its expression is different in different types of cancer. In view of the up- and downregulation of PTPRZ1 in different cancers, the pharmacological inhibition or activation of PTPRZ1 may be a promising strategy for tumors treatment. PTPRZ1 plays a role in cell proliferation, adhesion and migration, EMT, cancer stem cells, and treatment resistance by interacting or binding with several molecules [11]. It has been reported that PTN is one of the most important ligands of PTPRZ1. The interaction between PTPRZ1 and PTN increases the steady-state tyrosine dephosphorylation of *β*-catenin [26]. p120 is an adhesion junction protein, which plays a role in the adhesion and signal transduction between cells [27]. Attenuation of p120 expression may be a potential treatment strategy for pulmonary fibrosis [28]. According to the TCGA data, PTPRZ1 may be related to OS and DFS in various cancers, which suggests PTPRZ1 is involved in survival signals [29]. PTPRZ1 has an important impact on the survival of OSCC patients, and the risk of death within 5 years of positive PTPRZ1 patients is 8 times lower than that of patients with negative PTPRZ1 [30]. Knockdown of P120 promotes OSCC cell proliferation and tumor growth through C-*γ*1 signal transduction [31]. p-p120 promotes the progression and invasion of OSCC cells and may be a potential marker of OSCC disease [16]. As for the decrease of P120 and the increase of p-p120 expression level, this may be similar to the changes after *β*-catenin phosphorylation. Phosphorylation of *β*-catenin initiates degradation of the total *β*-catenin protein, resulting in a decrease in total protein content [32, 33]. In this paper, we found PTPRZ1, expression in OSF, and OSCC tissue was higher than that of normal oral mucosa tissue group, while p120 expression was lower. Besides, p-*β*-catenin was mainly expressed on the cell membrane, and PTPRZ1 and P–P120 were detected in all parts of cells with the same change trend. Pearson correlation coefficient analysis showed that PTPRZ1 was positively correlated with *β*-catenin and negatively correlated with p120. We found that the OSF malignant transformation is related to the PTPRZ1/*p*120/*β*-catenin axis.

Studies have shown that chronic oxidative stress leads to the amplification and overexpression of PTPRZ1 protein tyrosine phosphatase, thereby activating the *β*-catenin pathway [34]. In the rat renal cell carcinoma model, *β*-catenin pathway regulated by PTPRZ1 was the mediator of rat renal cell carcinoma carcinogenesis. *β*-catenin translocated to the nucleus and activated the downstream target genes [13]. PTPRZ1 promoted *β*-catenin tyrosine dephosphorylation and further increased the involvement of *β*-catenin in T cell factor (TCF) mediated transcription [35]. p120 was an upstream regulator of neurogenesis and cell cycle pathways in glioma patients and a predictor of poor clinical prognosis [36]. p120 could inhibit RhoA and stabilize the cadherin and *β*-catenin on the plasma membrane [37]. p120 stabilized the membrane association of *β*-catenin, thereby preventing the accumulation of *β*-catenin nuclei and the excessive activation of the WNT pathway during EMT [38]. We found that p-*β*-catenin was mainly expressed on the cell membrane, and PTPRZ1 and P–P120 were detected in all parts of cells with the same change trend. We found that PTPRZ1 could affect OSF carcinogenesis by regulating the phosphorylation of p120/*β*-catenin.

It was reported that *β*-catenin tyrosine phosphorylation led to a loss of intercellular adhesion, which in turn led to elevated cytoplasmic *β*-catenin levels, migrated to nucleus, and bound to transcription factor TCF/LEF. These led to increased MYC and cyclin D1 expressions, stimulated cell cycle, and proliferation [39]. PTPRZ1 knockdown could reduce the number of nuclear *β*-catenin and inhibit cell proliferation, as well as reduce target genes cyclin D1 and c-myc expressions in renal cancer [34]. Our results revealed that cyclin D1 and c-myc expressions were increased after overexpression of *β*-catenin. P120 and its phosphorylation of Tyr228 could inhibit the proliferation and invasion of colon adenocarcinoma cells [40]. Previous studies reported that increased expression of p120 isoforms 1A not only can upregulate E-cadherin and *β*-catenin but also can downregulate Rac1 activity and inhibit cell invasion. On the contrary, overexpression of p120 isoforms 3A could cause CDC42 inactivation and RhoA activation and has a small effect on invasion [41]. It has also been reported that deletion of p120 can inactivate RhoA but increase the activity of CDC42 and Rac1 and promote the proliferation and invasion of lung cancer cells [42]. We found that RhoA, Rac1, and CDC42 expressions increased after silencing p120. Through a series of cell function experiments, we found that PTPRZ1 overexpression promoted cell proliferation, migration, and invasion. Silencing p120 and overexpression of *β*-catenin can also promote cell proliferation, migration, and invasion. In nude mice tumor formation experiments, it was found that silencing PTPRZ1 can inhibit the development of OSCC. These showed that PTPRZ1/*p*120/*β*-catenin was related to the development process of OSF.

## 5. Conclusions

Our results showed that overexpression of PTPRZ1 could regulate p120/*β*-catenin phosphorylation to promote the malignant transformation of OSF. Our research provided ideas for the pathogenesis of OSF carcinogenesis. It was clear that the PTPRZ1/*p*120/*β*-catenin axis might serve as a potential therapeutic target and will help enrich new therapeutic strategies for OSF.

## Figures and Tables

**Table 1:** The primers used in this study.

Primer ID	5′-3′
PTPRZ1-F	TGCAGAGCTGTACTGTTGACTT
PTPRZ1-R	TGCCATCCTTTTCAGCAAGC
p120-F	ATGAGTGGTTCTCCAGAGGGA
p120-R	GCAGAGCAGAGCGGATGTAT
*β*-Catenin-F	ATTCTTGGCTATTACGACAGACT
*β*-Catenin-R	AGCAGACAGATAGCACCTT
RhoA-F	ACACACCAGGCGCTAATTCA
RhoA-R	CCCCAGAGCTATGCCAACAA
Cyclin D1-F	ACCTCTTCACCTTATTCATGGCT
Cyclin D1-R	GCCTTTCCCGACCCTGCTAC
Rac1-F	TGACCCTCTTTACCTCGCCCAC
Rac1-R	AACATCGTCAGCACTAGCACAG
CDC42-F	ACAAACAGAAGCCTATCACTCC
CDC42-R	CTGCGGCTCTTCTTCGGTTC
c-myc-F	ACACTAACATCCCACGCTCTG
c-myc-R	CGCATCCTTGTCCTGTGAGT
*β*-Actin-F	ACCCTGAAGTACCCCATCGAG
*β*-Actin-R	AGCACAGCCTGGATAGCAAC

## Data Availability

The data that support the findings of this study are available from the corresponding author upon reasonable request.
